# Worsening of chronic house-dust-mite-induced respiratory allergies: An observational survey in three European countries^☆^

**DOI:** 10.1016/j.waojou.2021.100563

**Published:** 2021-07-07

**Authors:** Pascal Demoly, Catherine Bos, Carmen Vidal

**Affiliations:** a Allergy Division, Pulmonology Department, Hôpital Arnaud de Villeneuve, University Hospital of Montpellier, Montpellier, France; b Sorbonne Université, UMR-S 1136 INSERM, IPLESP, Equipe EPAR, Paris, France; c Stallergenes Greer, Antony, France; d Allergy Service, Complejo Hospitalario Universitario de Santiago, Santiago de Compostela, Spain

**Keywords:** Respiratory allergy, House dust mite, Disease exacerbation, Worsening

## Abstract

**Background:**

Although respiratory allergies to house dust mites (HDMs) can often be controlled with symptomatic medications, some patients do not achieve satisfactory disease control.

**Objective:**

To assess fortnightly fluctuations (notably worsening and/or exacerbations) in disease parameters among patients taking only symptomatic medications for HDM allergy.

**Methods:**

In a 13-month, observational, multicenter survey of adults with a self-reported history of poorly controlled, moderate-to-severe, physician-diagnosed HDM respiratory allergy in France, Italy, and Spain, fortnightly telephone interviews were used to gather information on medication use, symptoms, the disease burden, and medical consultations from late May 2012 to early July 2013.

**Results:**

A total of 313 patients completed the study (*n* = 114 in Italy, 92 in France, and 107 in Spain). Although most participants reported improvements in symptoms, a substantial minority (ranging from 12% to 44% per fortnightly telephone interview in 2012 and from 16% to 37% in 2013) complained of worsening. A few study participants did not improve at any time in the study: 4% overall, and 2%, 2%, and 7% in Italy, France and Spain, respectively. A change in the weather and/or contact with other allergens were the most frequent self-reported reasons for worsening, although the answer “I don't know” was also prominent.

**Conclusion:**

In a 13-month survey of patients with HDM allergy in Italy, France, and Spain, the participants’ symptom status fluctuated significantly — illustrating the complexity of this condition. Although most participants reported improvements, the “never-improver” profile warrants further investigation. More prominence could be given to symptom control and a low exacerbation risk as treatment goals in allergic rhinitis.

## Introduction

Respiratory allergies, such as allergic rhinitis (AR) and allergic asthma, due to house dust mites (HDMs) affect more than 500 million people worldwide, albeit with significant geographical variations in exposure and seasonality.^1^, ^2^, ^3^, ^4^ Although the symptoms of HDM-induced respiratory allergy are rarely absent in sensitized individuals, the intensity varies over time as the indoor HDM populations and allergen levels fall or rise as a function of changes in the weather or the domestic environment.^5^, ^6^, ^7^, ^8^, ^9^ This is likely to be partly due to seasonal variations in domestic HDM populations (with peaks in September–November and March–May in the northern hemisphere^6^^,^^10^) and in the amount of time that people spend indoors (ie, most during the winter and less during the summer). Variations in co-sensitizing allergens (eg, pollen and mold) may also influence symptom intensities and exacerbations.^11^

In order to better characterize HDM-induced respiratory allergies from the patient's perspective, we performed a 13-month, observational, multicenter, Internet- and telephone-based survey of adult patients with a self-reported history of moderate-to-severe, poorly controlled, physician-diagnosed HDM respiratory allergy (but no other physician-diagnosed respiratory allergies) in 3 European countries (France, Italy, and Spain) from late May 2012 to early July 20, 13.^12^^,^^13^ Briefly, a total of 22 995 people were screened; 339 were included in the study, and 313 (n = 114 in Italy, 92 in France, and 107 in Spain; 67% females, with a predominance of young adults; mean age: 37.2 years) completed the fortnightly telephone interview phase and the post-inclusion questionnaire. The study generated a large amount of data, some of which has already been reported: the study's methodology, the participants' baseline demographic and clinical characteristics, the changes in symptom prevalence and intensity, the disease burden, and medication use.^12^^,^^13^

In summary, the fortnightly data revealed that in most participants, (1) symptoms peaked in early October and late May (coinciding with higher physician consultation rates), (2) nose and eye symptoms were more prevalent than chest and skin symptoms, and (3) there was an overall trend towards a reduction in symptom prevalence, symptom intensity, and disease burden over the survey period. Despite this overall trend, most participants fluctuated between symptom stability, worsening, and improvement. A small proportion of participants — “never-improvers” — even reported that their symptoms had never improved at any time in the survey. Here, we present the data on symptom worsening over the 13-month study period.

## Methods

The design, procedures, and inclusion and exclusion criteria of this observational, Internet- and telephone-based survey have been described in detail elsewhere but are summarized in Supplemental Fig. 1. In line with the respective legislations in France, Spain, and Italy on non-interventional, anonymous survey, regulatory approval from independent ethics committees was not required. The survey participants had provided their prior general consent to exploitation of the anonymized data. The study data were de-identified before analysis. Participants screened themselves for eligibility with a short Internet questionnaire and had to confirm that they had been diagnosed with HDM allergy by a physician. Participants received modest remuneration for their participation.

The study's inclusion criteria were as follows: age 18 years or over; moderate-to-severe symptoms of HDM allergy; at least 3 of the following symptoms: blocked nose, runny nose, itchy nose, difficulty breathing, cough, wheezing, sneezing, chest tightening, itchy eyes and tearing; physician-diagnosed allergy to HDM (and, in some individuals, other allergens); a positive skin prick test or a specific serum immunoglobulin E assay for HDM allergens; more severe allergic symptoms in September, October, November, or December; no previous or current allergy immunotherapy (AIT); use of at least one antihistamine or corticosteroid medication; symptoms not sufficiently controlled by current medication; a moderate to very strong impact of HDM allergy on quality of life.

After filling out a 28-question telephone-based post-inclusion questionnaire, the participants completed a 10-question fortnightly telephone-based interview from late May 2012 to early July 2013. Lastly, the participants completed a 24-question telephone-based final questionnaire at the end of the study. A descriptive analysis of the survey data was performed using SPSS software (version 15.0.1, IBM Corporation, Armonk, NY, USA). Quantitative parameters are expressed as the mean or the median and range, and qualitative parameters are expressed as the frequency (percentage).

## Results

A total of 22 995 individuals were screened; 339 of these (1.5%) were included and 313 (*n* = 114 in Italy, 92 in France, and 107 in Spain) completed the fortnightly study phase and the post-inclusion questionnaire.^12^^,^^13^ The baseline characteristics of the study population have been published in our initial report on the study but are summarized here for convenience (Supplemental Table 1).^12^^,^^13^ As reported previously, the majority of participants (61% in Italy, and 79% in France and Spain) reported having another physician-diagnosed respiratory allergy (mainly due to grass pollen, followed by weed pollen, pet dander, and tree pollen).^12^^,^^13^ For 85 participants (*n* = 44 in Italy, 19 in France, and 22 in Spain), HDM allergy was the only self-reported, physician-diagnosed allergy. Results for these “HDM-only” participants were processed separately.

### Improvements, worsening or stability in the symptoms of HDM allergy over a year-long period

**Fig. 1:**
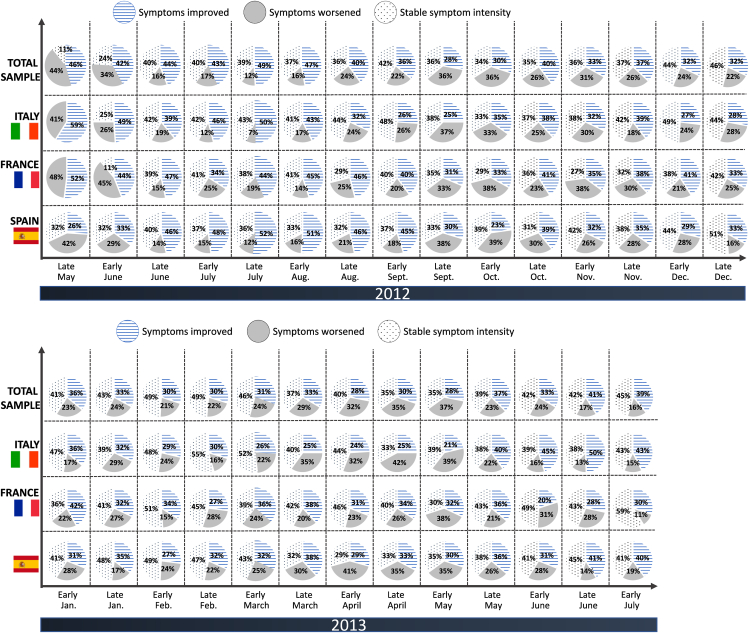
Fortnightly telephone interviews of study participants: the improvement, worsening or stability of allergy symptoms from late May to December 2012 (upper panel) and from January to July 2013 (lower panel) overall and in each study country

The overall time trends were similar for the fortnightly-rated prevalence of nasal, ocular, and respiratory symptoms: the prevalence fell during the summer of 2012, rose in the autumn (peaking in early October), fell over the winter, peaked again in the spring of 2013, and then fell over the summer, as previously reported.^12^^,^^13^ One of the fortnightly questions posed to each participant was “Overall, would you say that your symptoms have improved or worsened over the last couple of weeks?". Regardless of the increases or decreases in the prevalence of symptoms, a substantial minority of participants complained of worsened symptoms during the fortnightly questionnaires (ranging from 12% to 44% in 2012 and from 16% to 37% in 2013) (Fig. 1). It should be borne in mind that some participants reporting a worsening in one fortnightly survey reported stable symptoms or even an improvement in the next fortnightly survey. Logically, the proportion of participants reporting a worsening over the previous fortnight was lowest when the mean symptom prevalence decreased. The time trends in the proportion of worsened symptoms were similar in the 3 study countries. However, on the individual level, a small proportion of study participants (4% overall) did not improve at any time during the 13-month survey; the corresponding proportions of “never-improvers” in Italy, France, and Spain were 2%, 2%, and 7%, respectively. Conversely, a small proportion of study participants never worsened: 11% overall, and 12%, 6%, and 13% in Italy, France, and Spain, respectively.

**Fig. 2:**
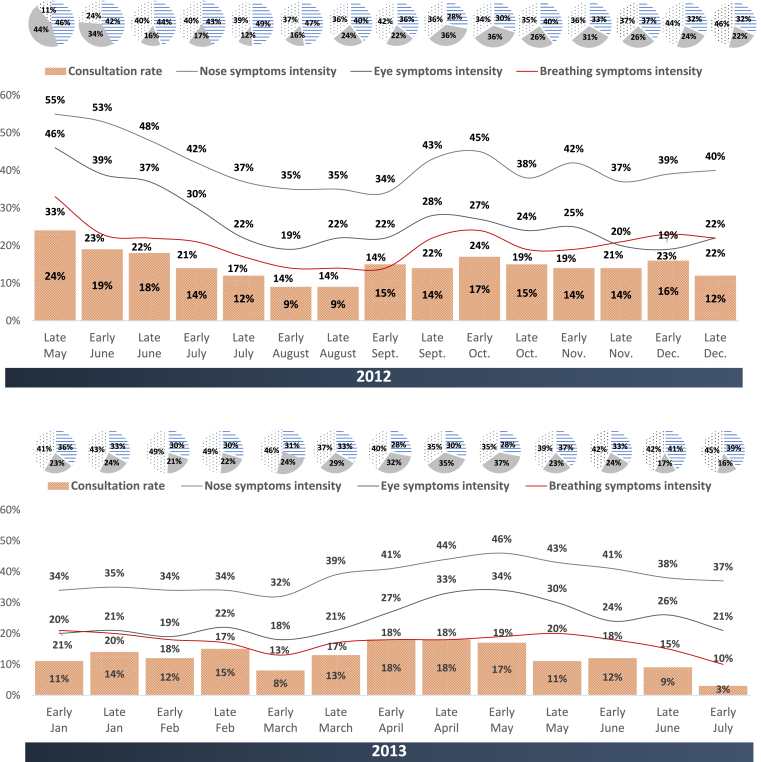
Fortnightly telephone interviews of study participants: the overall proportion of improvement, worsening or stability of allergy symptoms from late May to December 2012 (upper panel) and from January to July 2013 (lower panel), in comparison with the proportion of participants consulting a physician

Given that symptom worsening appeared to follow the same time trends as increases in symptom prevalence, it was not surprising to see that the proportion of participants consulting a physician also peaked when symptom worsening was most frequently reported in late spring and early autumn (Fig. 2).

### Reasons for symptom worsening in HDM allergy

**Fig. 3:**
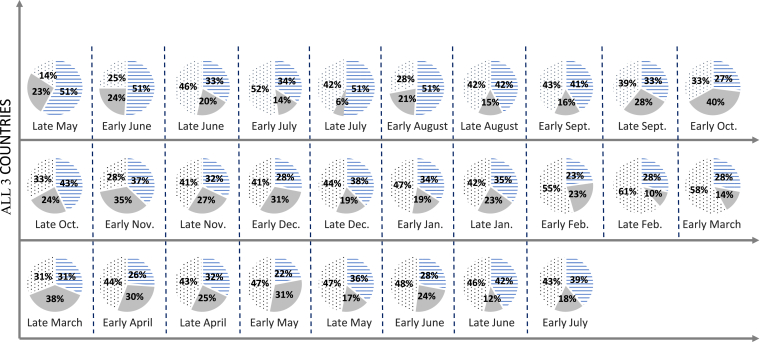
Fortnightly telephone interviews of “HDM-only” study participants (all three countries pooled): the improvement, worsening or stability of allergy symptoms from late May 2012 to July 2013

To first-take account of possible interference by concomitant allergen sensitizations in the seasonal trends observed for symptoms, the results for “HDM-only” participants were similar to those seen in the overall study population, with the highest proportions of worsening in autumn and spring (Fig. 3). In line with the overall results, a small proportion of the “HDM-only” participants (4% overall; 0%, 5%, and 10% in Italy, France, and Spain, respectively) did not improve at any time during the 13-month survey.

**Fig. 4:**
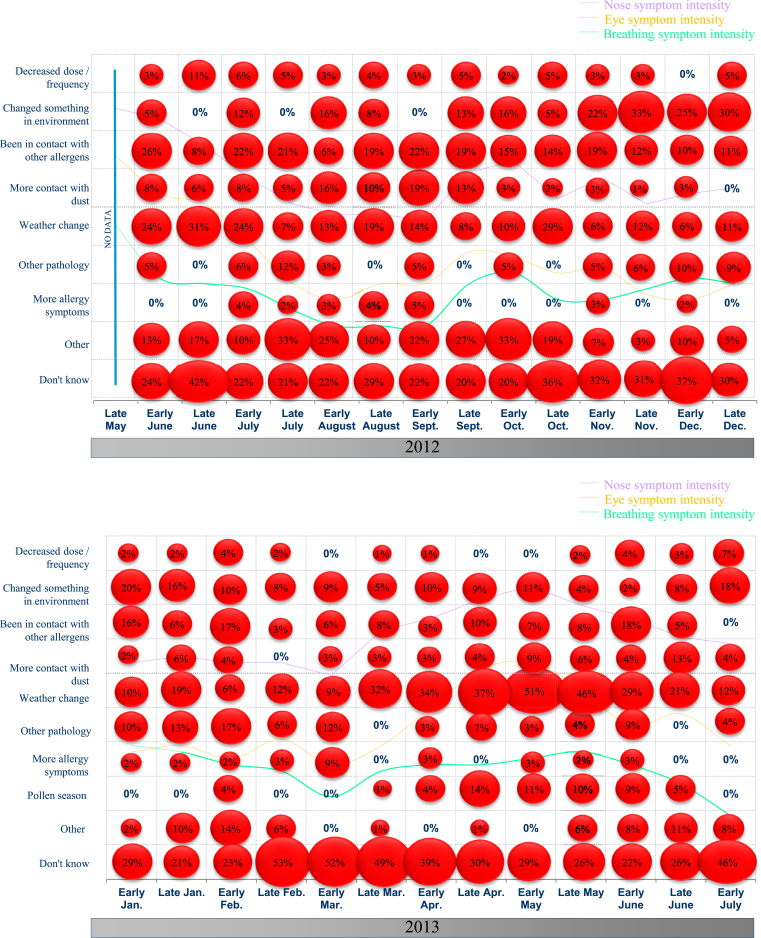
Fortnightly telephone interviews of study participants: the stated reasons for the worsening of allergy symptoms from late May to December 2012 (upper panel) and from January to July 2013 (lower panel)

When “worseners” were then asked why their symptoms had worsened, they could not always put forward a single, unambiguous reason. A change in the weather and/or contact with other allergens were the most frequently stated possible reasons (Fig. 4). However, in some fortnightly surveys, the most frequent answer was “I don't know” – which was also the most frequent answer from participants whose symptoms had improved (data not shown). These overall results were confirmed in the “HDM-only” population; “I don't know” was the most frequent answer with regard to why the allergy symptoms had worsened in the previous fortnight.

## Discussion

In a 13-month, observational, multicenter survey of adult patients with a self-reported history of moderate-to-severe, poorly controlled, physician-diagnosed HDM respiratory allergy (but no other physician-diagnosed respiratory allergies) in 3 European countries (France, Italy, and Spain), we found that (1) at each fortnightly survey, a substantial minority of participants complained of worsened symptoms, and (2) a small proportion of study participants did not improve at any time during the 13-month survey. These participants were probably those who used the most rescue medications and/or more frequently made unscheduled physician visits because of unexpected, troublesome symptoms. Our results also raise the issue of AR exacerbations, which are harder to define than asthma exacerbations. Many definitions of asthma exacerbations have been used, including patient-reported increases in symptom severity, reductions in air flow, increased medication use, and even hospital or emergency room admissions. The management of exacerbations is a longstanding, clinically important topic in the field of asthma.^14^ Recent clinical trials in AR have shown the benefit (vs. placebo) of a 12 standardized quality (SQ) HDM sublingual AIT (sublingual immunotherapy [SLIT]) tablet or a 300 index of reactivity (IR) HDM SLIT tablet on rhinitis exacerbations (defined as a high total combined score, or a high symptom score with at least 1 symptom that was hard to tolerate, interfered with activities of daily living and/or sleeping).^15^^,^^16^

The literature data show that the exacerbation of respiratory allergy symptoms is 1 of 3 most common indications for short (<30-day) courses of orally administered corticosteroids.^17^ However, even these short courses are associated with a two-to five-fold elevation in the incidence of acute adverse events that result in major morbidity and mortality (sepsis, venous thromboembolism, and fractures). Given that the corticosteroid-associated risk is dose-dependent and cumulative, it makes sense to avoid exacerbations and thus not to have to treat them.^18^

One way of reducing AR exacerbations may include the adoption of a stepwise, disease-control-based treatment approach based partly on the endotype (ie, the pathophysiological disease subtypes defined according to the medical history, physical examinations, *in vivo*/*in vitro* testing, etc), as has been introduced for asthma.^19^ In summary, treatment level 1 for AR is based on a second-generation oral antihistamine, level 2 moves to an intranasal corticosteroid, level 3 combines an intranasal corticosteroid with other medications, and level 4 is the specialist-only prescription of omalizumab in severe cases of severe rhinitis with concurrent asthma.^19^ This approach is also in line with the “step-up/step-down” clinical decision algorithm developed through the ARIA-GRADE and MACVIA initiatives, in which intranasal corticoids are initiated more readily (ie, level 1) as a function of the patient’ symptoms measured on a 0-to-10 visual analog scale (VAS) but are withdrawn (stepped down) once the VAS score falls below 5.^20^^,^^21^

Allergen immunotherapy should always be considered as the need for better disease control increases; as mentioned above, administration of HDM SLIT was associated with a lower probability of rhinitis exacerbations and a greater probability of mild symptom days.^16^ For pollen aeroallergens, exacerbations are most likely at the peak of the pollen season. A five-grass-pollen SLIT tablet was associated with significantly improved quality of life (according to the mean Rhinoconjunctivitis Quality of Life Questionnaire score) during the peak of the pollen season.^22^

Why, then, do the symptoms of HDM respiratory allergy fluctuate on such a short (week-to-week) timescale? With regard to other allergens, it has been reported that clinical exacerbations of asthma, AR, and sinusitis, and levels of pollen and mold allergens are associated with weather changes, such as strong "El Niño" events^11^ and devastating “thunderstorm asthma” events.^23^ Likewise, asthma-related visits to the emergency department are correlated with pollen counts.^24^^,^^25^ As mentioned above, there are seasonal variations in domestic HDM populations and in levels of associated allergens.^6^^,^^10^ However, these events typically happen on the timescale of years or months, rather than weeks. To the best of our knowledge, the seasonal variation of HDMs in France, Italy, and Spain has not been extensively characterized – in contrast to the Americas and Australia. We only found 1 study performed in Spain and 2 performed in France.^26^, ^27^, ^28^ However, all 3 studies highlighted seasonal variations, with higher levels of allergen in the spring and especially the autumn.^26^, ^27^, ^28^

More generally, the question of “worseners” is linked to efforts to explain why some patients respond to AIT and/or pharmacotherapy (“responders”) and others do not (“non-responders”). Researchers have sought to identify biochemical and demographic factors that predict a poor response or a good response to AIT and/or pharmacotherapy. For example, the Jakalski et al retrospective analysis of 1624 patients with AR found that AIT was less likely to be effective in polysensitized individuals and in those with a prolonged duration of AR prior to treatment initiation.^29^ Similarly, Qi et al classified 284 patients having received subcutaneous AIT for HDM-induced AR as responders or non-responders.^30^ The researchers found that a better response was associated with a short duration of allergy, a higher baseline symptom score, and local reactions. With regard to biomarkers, the analysis of peripheral blood mononuclear cells sampled from patients during a double-blind, placebo-controlled clinical trial of grass pollen SLIT tablets found that levels of complement component 1Q and stabilin 1 were higher in clinical AIT responders than in AIT non-responders or placebo-treated patients^31^ — suggesting that the treatment response has a constitutive (potentially genetic) basis. Other candidates for response markers include CD141, GATA3, RIPK4, C1QA, and FcγRIIIA on or in peripheral blood mononuclear cells.^32^ Lastly, nonresponse to treatment might also be linked to pathological factors or comorbidities such as chronic rhinosinusitis with or without nasal polyposis, or nasal septum deviation; we did not collect data on these variables.

This self-reported, observational, Internet- and telephone-based survey had several limitations.^12^^,^^13^ Firstly, survey participants were not examined by a study investigator, and so the HDM allergy, any other potential allergies or sensitizations, and the level of disease control were not confirmed clinically. However, to be included in the study, a potential participant had to state that (i) he/she suffered from HDM allergy, (ii) he/she had consulted a specialist physician for the HDM allergy, and (iii) the specialist physician had confirmed the diagnosis of HDM allergy. Secondly, over two-thirds of the participants in this real-life study reported that they had been diagnosed with 1 or more other allergies, apart from HDM allergy, and so we cannot rule out effects of possible concomitant allergies or even viral respiratory tract infections;^33^ this again is an inherent limitation of self-reporting. However, all participants only reported a single (HDM-induced) physician-diagnosed allergy, and the results for participants sensitized only to HDM allergens (the “HDM-only" group) mirrored those in the overall population. Thirdly, women and young adults were probably over-represented in the study population, as these groups are more likely to be Internet users.^34^ Fourthly, the participants’ data were recorded with custom questionnaires rather than psychometrically validated tools. Fifthly, the fortnightly telephone interviews suffered from a highly variable completion rate (which was relatively high on averaged — 75% — but ranged from 29% to 97%, depending on the country and the period), with the lowest values during holiday periods. Sixthly, indoor HDM allergen levels and outdoor pollen levels were not measured for cost reasons.^33^ Lastly, participation in the study itself (notably through the fortnightly questionnaires) might have induced a decrease in the symptoms experienced (ie, a type of placebo effect), as has been seen in clinical trials in the field of AR.^35^ Seventhly, the design of our purely descriptive survey ruled out a robust statistical analysis, and so we were unable to say whether or why (for example) the proportion of nonresponders was truly higher in Spain (7%) than in France and Italy (2% each).

The study's strengths included (i) its international, multicenter, longitudinal design, (ii) a relatively large number of participants, and (iii) detailed long-term data collection in real life.

## Conclusion

In a 13-month, international, multicenter survey of patients with a self-reported history of moderate-to-severe, poorly controlled, HDM-induced AR and asthma, we found that trends towards symptom stability, worsening or improvement were irregular and not clearly linked to changes in the participants’ circumstances. Future clinical research could usefully address the relationships between AR exacerbations, symptom profiles and non-responder status, and disease-related and treatment-related factors. As is already the case for the long-term management of asthma, it is high time to add symptom control and a reduction in the exacerbation risk as treatment goals in AR.

## Abbreviations

AIT: allergen immunotherapy; AR: allergic rhinitis; ENT: ear, nose and throat; FP: family physician; HDM: house dust mite; IR: index of reactivity; SLIT: sublingual allergen immunotherapy.

## Financial support

The study was funded by an unrestricted grant from Stallergenes Greer (Antony, France). Through one of its employees at the time (Catherine Bos), the funding source (Stallergenes Greer, Antony, France) was involved in analysis and interpretation of data, the writing of the report, and the decision to submit the article for publication.

## Conflict of interest disclosure

Dr. Demoly reports grants from ALK, Stallergenes Greer, AstraZeneca, ThermoFisher Scientific, Ménarini, Bausch & Lomb, GSK, personal fees from Sanofi Regeneron, outside the submitted work. Catherine Bos was an employee of Stallergenes Greer at the time when the study was performed. Carmen Vidal has nothing to disclose.

## Agreement to publish the work

All the authors have given final approval of the version to be published.

## Statement of contribution to the work

All authors made substantial contributions to study design and data collection and interpretation. Likewise, all authors were all involved in drafting the manuscript and revising it critically for important intellectual content.

## Ethics statement

In line with the respective national legislations, specific ethical and regulatory approval from institutional review boards or health authorities was not required for this non-interventional, phone-/Internet-based survey. The survey participants provided their prior general consent to exploitation of anonymized personal data.

## Editorial policy confirmation and agreement

The authors confirm that their manuscript is original, has not been published before, is not currently being considered for publication elsewhere, and has not been posted to a preprint server.

## Availability of data and material

The proprietary datasets generated and/or analyzed during the current study are not publicly available but are available from the corresponding author on reasonable request.

## Appendix ASupplementary data

Supplemental Fig. 1Study timeline

Supplemental Table 1Characteristics of the survey population, according to the post-inclusion questionnaire
