# Supplementary figures and images for: Worsening of chronic house-dust-mite-induced respiratory allergies: An observational survey in three European countries
Source: World Allergy Organ J. 2021 Jul 7;14(7):100563. doi: 10.1016/j.waojou.2021.100563 (PMC8271175; doi:10.1016/j.waojou.2021.100563)

Supplemental Fig. 1

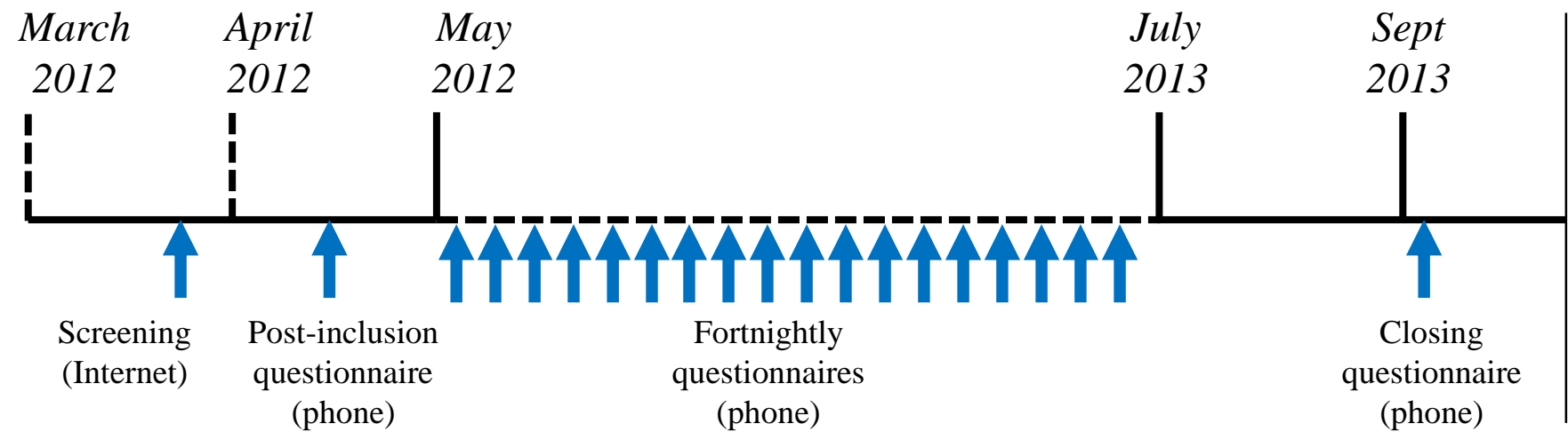

Supplement: Supplementary file 1 — Supplemental Fig. 1Study timeline [file mmc1.pdf]
