# Supplementary material for: Worsening of chronic house-dust-mite-induced respiratory allergies: An observational survey in three European countries
Source: World Allergy Organ J. 2021 Jul 7;14(7):100563. doi: 10.1016/j.waojou.2021.100563 (PMC8271175; doi:10.1016/j.waojou.2021.100563)
Supplement: Supplementary file 2 — Supplemental Table 1Characteristics of the survey population, according to the post-inclusion questionnaire [file mmc2.pdf]

Supplemental Table 1. Characteristics of the survey population, according to the post-inclusion questionnaire [12].

	Italy	France	Spain
Number of participants (*n* = males/females), % female	114 (36/78), 68%	92 (27/65), 72%	107 (41/66), 61%
Age (years) mean, median [range]	37.5, 36 [18–63]	35.8, 36 [18–62]	38.2, 37 [18–68]
Time since first symptoms (years) mean, median [range]	15.0, 16 [1–45]	17.8, 18 [1–51]	17.3, 13 [1–40]
Time interval between first symptoms and consultation with a specialist (months) mean, median [range]	18.9, 4 [0.25–588]	28.4, 6 [0.25–360]	20.3, 11 [0.25–156]
Other self-reported allergies (% of patients):			
grass pollen	79	67	67
Parietaria pollen	57	37	9
cat dander	49	51	41
dog dander	31	26	29
olive pollen	27	41	35
birch pollen	27	44	7
molds	24	29	35
cypress pollen	21	48	9
none (i.e. HDMs only)	39	21	21
Proportion of patients having consulted the following physicians, % (mean number of visits per year)			
FP	92 (3.4)	91 (3.0)	70 (3.9)

allergist	87 (2.0)	83 (1.7)	70 (1.4)
ENT specialist	27 (2.3)	27 (2.3)	15 (1.7)
dermatologist	24 (1.6)	20 (1.7)	8 (2.6)
pulmonologist	22 (1.5)	32 (1.6)	8 (6.2)
pediatrician	14 (6.8)	9 (1.0)	7 (n.a.)
other	6 (4.0)	2 (1.5)	4 (1.0)
Proportion of patients having consulted the following combinations of physicians, %			
FP only	17	9	5
allergist only	18	4	3
FP + allergist	33	25	31
FP + another specialist	7	7	5
FP + two specialists	11	29	26
FP + three or more specialists	4	22	28
Prevalence of co-morbidities, % of patients			
sinusitis	36	53	26
otitis	13	18	9
conjunctivitis	44	43	33
headache	69	62	69
Time having used symptomatic medications, % of patients			
less than 2 years	7	3	3
2–5 years	35	28	21
6–10 years	22	39	30
11–20 years	21	23	27
more than 20 years	16	7	15
Degree of disease control, % of patients			

Totally controlled	11	4	10
Well controlled	43	53	48
Moderately controlled	42	34	33
Poorly controlled	4	7	7
Not controlled at all	0	2	3
Proportion of patients suffering from symptoms for more than 4 days in a week, %	70	58	54
Proportion of patients taking medications, %			
antihistamines	38	40	34
nasal corticoids	7	12	10
inhaled corticoids	3	3	9
bronchodilators	7	12	15
inhaled corticoids + bronchodilator	5	7	5
oral or topical corticoids	12	3	4
eye drops	4	6	4
leukotriene receptor antagonists	2	4	3

HDM: house dust mite; FP: family physician; ENT: ear, nose and throat; n.a. not available
